# Ultrasound-Assisted Extraction, Antioxidant and Anticancer Activities of the Polysaccharides from *Rhynchosia minima* Root

**DOI:** 10.3390/molecules201119734

**Published:** 2015-11-23

**Authors:** Xuejing Jia, Chao Zhang, Jie Hu, Muxue He, Jiaolin Bao, Kai Wang, Peng Li, Meiwan Chen, Jianbo Wan, Huanxing Su, Qingwen Zhang, Chengwei He

**Affiliations:** State Key Laboratory of Quality Research in Chinese Medicine, Institute of Chinese Medical Sciences, University of Macau, Macau 999078, China; jiaxjsicau@gmail.com (X.J.); 13596008379@126.com (C.Z.); hujie0811@163.com (J.H.); mb45808@umac.mo (M.H.); fish1012@hotmail.com (J.B.); wangkai6648@gmail.com (K.W.); pengli@umac.mo (P.L.); MWChen@umac.mo (M.C.); JBWan@umac.mo (J.W.); HuanxingSu@umac.mo (H.S.); qwzhang@umac.mo (Q.Z.)

**Keywords:** *Rhynchosia minima* root, polysaccharide, ultrasound-assisted extraction, antioxidant activity, anticancer activity

## Abstract

Box-Behnken design (BBD), one of the most common response surface methodology (RSM) methods, was used to optimize the experimental conditions for ultrasound-assisted extraction of polysaccharides from *Rhynchosia minima* root (PRM). The antioxidant abilities and anticancer activity of purified polysaccharide fractions were also measured. The results showed that optimal extraction parameters were as follows: ultrasound exposure time, 21 min; ratio of water to material, 46 mL/g; ultrasound extraction temperature, 63 °C. Under these conditions, the maximum yield of PRM was 16.95% ± 0.07%. Furthermore, the main monosaccharides of purified fractions were Ara and Gal. PRM3 and PRM5 exhibited remarkable DPPH radical scavenging activities and reducing power *in vitro*. PRM3 showed strong inhibitory activities on the growth of MCF-7 cells *in vitro*. The above results indicate that polysaccharides from *R. minima* root have the potential to be developed as natural antioxidants and anticancer ingredients for the food and pharmaceutical industries.

## 1. Introduction

Ultrasound-assisted extraction, by means of ultrasonic vibration, can facilitate the release of components from sample matrices [1]. Due to the cavitation in a strong acoustic field, this widely used extraction technique has a lot of advantages, such as taking less extraction time, synchronously processing a large number of extraction samples, and increasing the diffusion rate [2,3]. Response surface methodology (RSM), a validated collection of statistical and mathematical techniques, is commonly used to optimize and evaluate complex experimental process factors and their interactions [4]. Furthermore, RSM needs less experimental trials and labor than many other optimized approaches [5]. The Box-Behnken design (BBD), one of the most used RSM methods, plays a pivotal role in arranging and interpreting the optimal experiments. Currently, multiple literatures have reported that ultrasound-assisted extraction with RSM is commonly applied in analyzing bioactive components of plants, such as *Chrysanthemum morifolium* flower heads [6], *Ligusticum* rhizomes [7], sweet basil [8], and sugar beet [9].

*Rhynchosia minima* root, a traditional medicinal herb, is used to alleviate skin abscesses, upper respiratory infections, or joint pains. Our previous results indicated that its polysaccharides showed strong anticancer activity against human lung cancer A549 and liver cancer HepG2 cells in a dose-dependent manner [10]. In order to further develop and explore this valuable resource, there is a pressing demand to obtain extensive crude polysaccharides in a short span of time. To reduce time and energy consumption, thus, it is urgent to optimize the extraction conditions for isolating the polysaccharides from *R. minima* roots. In the present study, we optimized the ultrasound-assisted extraction parameters based on BBD, including ultrasound exposure time, ratio of water to material and ultrasound extraction temperature, to maximize the polysaccharide yield of *R. minima* roots. In addition, the obtained crude polysaccharides were isolated, purified and lyophilized. Antioxidant activity and anticancer activity of purified fractions were also evaluated *in vitro*.

## 2. Results and Discussion

### 2.1. Single Factor Experimental Analysis

**Figure 1:**
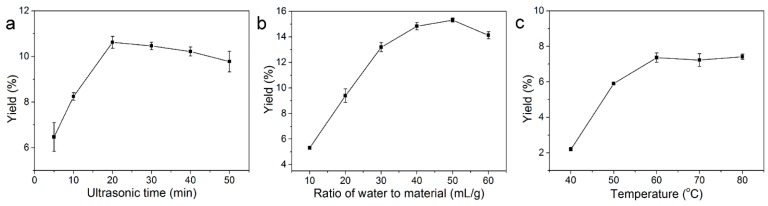
Effect of different extraction factors ((**a**) ultrasound exposure time, min; (**b**) ratio of water to material, mL/g; (**c**) ultrasound extraction temperature, °C) on the extraction yield of PRM. The effects of ultrasound exposure time, ratio of water to material and ultrasound extraction temperature were first studied by a single-factor design as follows: only one factor was changed while the other factors were kept constant in each independent experiment. The effect of each factor was evaluated by determining their yield of PRM.

The effects of various extraction parameters, including ultrasound exposure time, ratio of water to material and ultrasound extraction temperature, on the yield of polysaccharides were measured using the one variable at a time approach [11]. The effect of ultrasound exposure time on the yield of PRM is shown in Figure 1a. Various times (5, 10, 20, 30, 40 and 50 min) were examined, while other extraction variables were kept as follows: ratio of water to material, 50 mL/g; ultrasound extraction temperature, 50 °C. The yield of PRM increased rapidly for increasing ultrasound exposure times ranging from 5 to 20 min. Thereafter, however, it reached a plateau with a slight decrease, which might be due to polysaccharide degradation after extended ultrasonic-assisted extraction [12]. To avoid energy consumption and reduce the extraction time, 20 min was selected as the optimum ultrasound exposure time for PRM production.

The effect of ratio of water to material on the yield of PRM is shown in Figure 1b. The other variables were fixed as follows: ultrasound exposure time, 20 min; ultrasound extraction temperature, 50 °C. It was clear that PRM yield rose and peaked at the ratio of 40 mL/g. Therefore, 40 mL/g was chosen as the optimum ratio of water to material.

The effect of ultrasound extraction temperature on the yield of PRM was investigated with the ratio of water to material, 40 mL/g; ultrasound exposure time, 20 min. As shown in Figure 1c, the yield of PRM increased sharply when the ultrasound extraction temperature rose from 40 to 60 °C, and leveled off with further temperature increases. Thus, the optimal ultrasound extraction temperature was 60 °C.

### 2.2. Fitting the Models

According to the single factor experiment results, BBD with seventeen runs was applied to optimize the three independent extraction variables, including ultrasound exposure time (A); ratio of water to material (B); and ultrasound extraction temperature (C). Table 1 showed the BBD matrix and the experimental PRM yields. Regression analysis revealed that the yield of PRM (Y) and three independent variables could be expressed by the following second-order polynomial equation:

Y = 16.46 + 0.62A + 0.75B + 0.96C − 0.25AB + 0.33AC − 0.16BC − 4.52A^2^ − 0.63B^2^ − 1.56C^2^ (1)

where Y is the experimental yield of PRM; A, B and C are the coded factors of ultrasound exposure time, ratio of water to material and ultrasound extraction temperature, respectively.

**Table 1:** BBD with the experimental and predicted values for the yield of PRM.

Run	Coded			Actual			Yield (%)	Predicted Yield (%)
	A ^a^	B ^a^	C ^a^	A ^a^	B ^a^	C ^a^		
1	0	0	0	20	40	60	16.82	16.46
2	0	0	0	20	40	60	16.71	16.46
3	−1	0	1	10	40	70	10.08	10.40
4	1	0	−1	30	40	50	10.01	9.70
5	−1	−1	0	10	30	60	9.67	9.70
6	1	1	0	30	50	60	12.46	12.43
7	0	−1	1	20	30	70	14.99	14.65
8	1	0	1	30	40	70	12.29	12.29
9	0	1	1	20	50	70	15.80	15.83
10	0	0	0	20	40	60	16.66	16.46
11	−1	1	0	10	50	60	12.03	11.68
12	0	−1	−1	20	30	50	12.43	12.41
13	0	0	0	20	40	60	15.62	16.46
14	1	−1	0	30	30	60	11.09	11.43
15	0	1	−1	20	50	50	13.87	14.21
16	−1	0	−1	10	40	50	9.13	9.13
17	0	0	0	20	40	60	16.02	16.46

^a^ A-Ultrasound exposure time (min); B-Ratio of water to material (mL/g); C-Temperature (°C).

Statistical analysis of this model was performed using one-way ANOVA. The results (Table 2) indicated that the model was significant (*p* < 0.0001), but lack of fit was not significant (*p* > 0.05), which revealed that this model could be well fitted to the experimental data and appropriately describe the relationship between the extraction variables and the response value [6].

**Table 2:** ANOVA results for the yield of PRM.

Source	Sum of Squares	df ^a^	Mean Square	*F*-Value	*p*-Value
Model	116.50	9.00	12.94	51.44	<0.0001
A ^b^	3.05	1.00	3.05	12.12	0.0102
B ^b^	4.46	1.00	4.46	17.71	0.0040
C ^b^	7.45	1.00	7.45	29.60	0.0010
AB	0.24	1.00	0.24	0.96	0.3609
AC	0.44	1.00	0.44	1.75	0.2276
BC	0.10	1.00	0.10	0.39	0.5537
A^2^	84.32	1.00	84.32	335.12	<0.0001
B^2^	1.43	1.00	1.43	5.67	0.0488
C^2^	9.63	1.00	9.63	38.28	0.0005
Residual	1.76	7.00	0.25		
Lack of fit	0.67	3.00	0.22	0.82	0.5481
Pure error	1.09	4.00	0.27		
Correlation total	118.26	16.00			
R^2^ = 0.9851	Adj-R^2^ = 0.9660		CV = 3.78%		

^a^ df is the degree of freedom; ^b^ A-Ultrasound exposure time (min); B-Ratio of water to material (mL/g); C-Temperature (°C).

The coefficient of variation (CV) was 3.78, which indicated that this model had good precision and reliability. The coefficient of determination (R^2^) and adjusted coefficient of determination (Adj-R^2^) were 0.9851 and 0.9660, respectively, which indicated that this polynomial model had adequate accuracy and general applicability [13]. In this regard, the three independent variables (A, B and C) and two quadratic terms (A^2^ and C^2^) were significant, but the three interactions (AB, AC and BC) were not significant.

### 2.3. Response Surface Analysis

**Figure 2:**
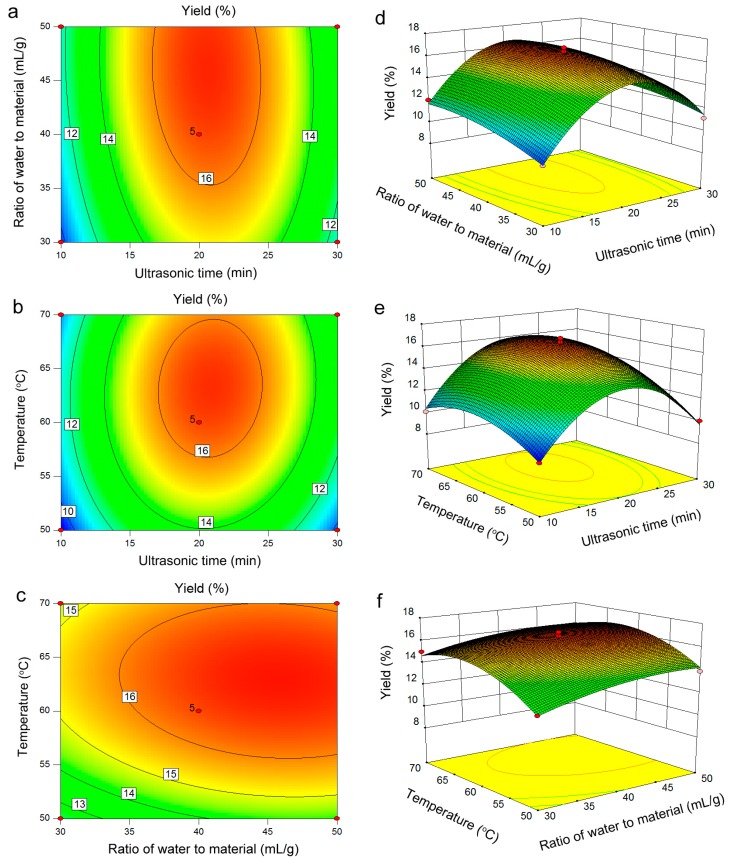
Contour plots (**a**–**c**); and 3D response surface plots (**d**–**f**) showing the interaction effects on the extraction yield of PRM; (**a**,**d**), ratio of water to material and ultrasound exposure time; (**b**,**e**), ultrasound extraction temperature and ultrasound exposure time; (**c**,**f**), ultrasound extraction temperature and ratio of water to material.

The mutual interactions of independent and dependent variables on the yield of PRM could be visualized by three dimensional (3D) response surface plots and two dimensions contour plots (Figure 2). Each 3D plot represented the number of combinations of the two-test variables. The shapes of contour plots represented the significance of the mutual interactions. Circular contour plots indicated that the interactions were non-significant, whereas elliptical contours demonstrated the interactions were significant [14].

From these response surface plots (Figure 2) and one-way ANOVA analysis (Table 2), it was evident that ultrasound extraction temperature was the most significant factor affecting the yield of PRM, followed by the ratio of water to material and ultrasound exposure time. The suitability of the model quadratic equations for predicting the optimal response values was checked using the selected conditions. The optimal conditions were adjusted as followed: ultrasound exposure time, 20.6 min; ratio of water to material, 45.5 mL/g; ultrasound extraction temperature, 62.9 °C. Under these conditions, the predicted yield was 16.82%. However, considering the practical operability, the operated conditions were modified as follows: ultrasound exposure time, 21 min; ratio of water to material, 46 mL/g; ultrasound extraction temperature, 63 °C. Under the above modified conditions, the yield of PRM was 16.95% ± 0.07% (*n* = 3), which agreed well with the predicted yield and simultaneously confirmed this model was satisfactory and valid.

### 2.4. Monosaccharide Composition of Purified Fractions

**Figure 3:**
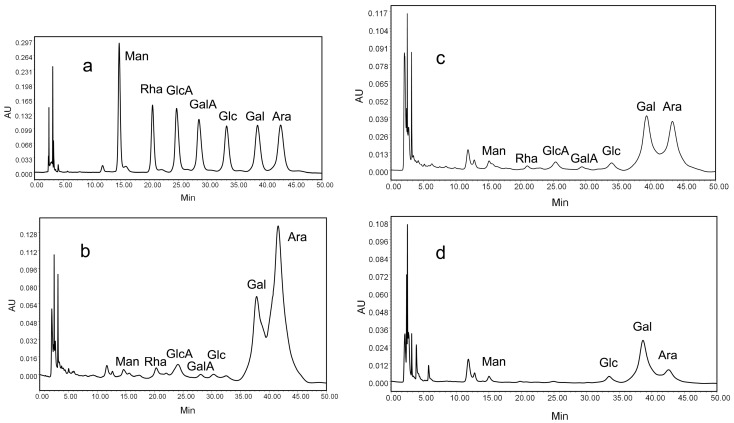
HPLC analysis of monosaccharide composition of standard (**a**); PRM1 (**b**); PRM3 (**c**); and PRM5 (**d**). The three polysaccharides were hydrolyzed with trifluoroacetic acid, labeled with PMP, and analyzed with a Waters e2695 HPLC instrument.

The HPLC results showed that each detectable monosaccharide had a single sharp peak (Figure 3a). PRM1, PRM3 and PRM5 had similar monosaccharide compositions but with different molar ratios. The molar ratios of Man, Rha, GlcA, GalA, Glc, Gal, Ara in PRM1 and PRM3 were 0.5:0.6:1.4:0.2:0.3:15.8:36.7 (Figure 3b) and 0.5:0.4:1.4:0.5:2.3:26.7:23.9 (Figure 3c), respectively, while the molar ratio of Man, Glc, Gal, Ara in PRM5 was 1.9:3.0:45.7:5.4 (Figure 3d). Gal and Ara were the main components of the three purified polysaccharides, which was in line with our previous results [10].

### 2.5. In Vitro Antioxidant Activity of Purified Fractions

**Figure 4:**
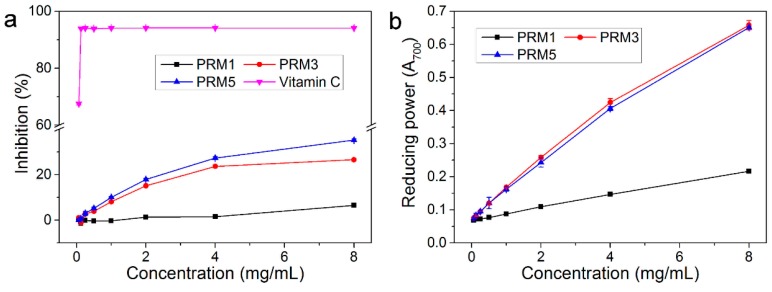
*In vitro* antioxidant activities of PRM1, PRM3 and PRM5. (**a**) DPPH radical scavenging activity; (**b**) Reducing power.

In order to determinate the antioxidant activity of PRM1, PRM3 and PRM5, the DPPH radical scavenging activity was tested. The results are shown in Figure 4a. PRM3 and PRM5 exhibited stronger scavenging activity than PRM1, while they all were far lower than that of vitamin C. At 8 mg/mL, the percentages of scavenging for PRM1, PRM3 and PRM5 were 6.55%, 26.53% and 35.13%, respectively. In addition, the IC_50_ values of PRM1, PRM3 and PRM5 were 343.24, 24.32 and 13.98 mg/mL, respectively. Thi**s** results indicated that PRM5 could supply more hydrogen atoms than PRM1 and PRM3 [15].

The reducing power assay reveals the electron donating activity and can be used to evaluate the potential antioxidant activity of polysaccharide fractions [16,17]. As shown in Figure 4b, the reducing abilities of all fractions increased with the increasing concentration. In addition, PRM3 and PRM5 displayed better reducing capacity than PRM1. At 8 mg/mL, the reducing power of these fractions were in the order of PRM3 ≈ PRM5 > PRM1.

The above-mentioned results indicated that all purified polysaccharide fractions had strong dose-dependent antioxidant activities. PRM5 and PRM3 exhibited stronger antioxidant activities than PRM1. This might be attributed to the fact that PRM5 and PRM3 possessed high uronic acid contents and relatively low molecular weight [10,18].

### 2.6. In Vitro Anticancer Activity of Purified Fractions

**Figure 5:**
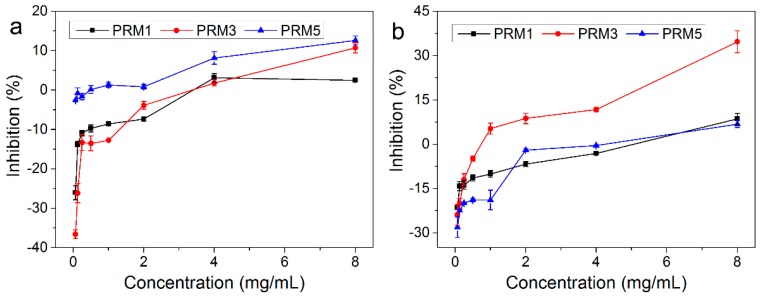
*In vitro* anticancer activities of PRM1, PRM3 and PRM5 against human breast cancer MCF-7 cells determined by MTT method. (**a**) Treatment for 24 h; (**b**) Treatment for 48 h.

The *in vitro* growth inhibitory effects of PRM1, PRM3 and PRM5 against human breast cancer MCF-7 cells were studied using the MTT assay. The MCF-7 cells were treated with various polysaccharide concentrations (0.0625, 0.125, 0.25, 0.5, 1, 2, 4 and 8 mg/mL for PRM1, PRM3 and PRM5) for 24 h and 48 h. In Figure 5, all three polysaccharide fractions showed a significant growth inhibitory effect in a dose-dependent manner. After treatment with polysaccharide fractions for 24 h, only PRM5 showed a certain degree of growth inhibition against MCF-7 cells. However, after treatment for 48 h, the inhibitory percentage of PRM5 roughly remained the same as the results of 24 h. At 8 mg/mL, after treatment for 48 h, the anticancer abilities of fractions against MCF-7 cells were in the order of PRM3 > PRM5 ≈ PRM1. PRM3 exhibited the best anticancer activity and its IC_50_ value was 15.61 mg/mL.

It was well-known that the anticancer activity of polysaccharide might be associated with its monosaccharide composition, molecular weight, and structural conformation [19,20]. Our previous study observed that PRM3 with moderate uronic acid contents and low molecular weight could exhibit potent anticancer activities *in vitro* [10]. In this study, PRM3 also possessed strong antioxidant activity *in vitro*, which indicated that PRM3 was a powerful source of hydrogen atoms and was good at transferring electrons [15,21,22]. However, the mechanism of the *in vitro* antioxidant activity and anticancer activity of these polysaccharide fractions is still not clear, and needs to be further investigated.

## 3. Experimental Section

### 3.1. Materials and Reagents

*R. minima* roots were purchased from an apothecary shop (Pingtan County, Fujian, China). The roots were dried, crushed and grounded into fine powder, and then stored in a desiccator to prevent any contact of moisture before experiments. 3-(4,5-Dimethyl-2-thiazolyl)-2,5-diphenyl-2-*H*-tetrazolium bromide (MTT), 1,1-diphenyl-2-picryhydrazyl (DPPH), DEAE Cellulose-52 and Sephadex G-150 were purchased from Sigma-Aldrich Co., Ltd. (St. Louis, MO, USA). All other reagents were of analytical grade and purchased from Aladdin Industrial Corporation (Shanghai, China).

### 3.2. Optimal Extraction of PRM

The dried *R. minima* roots were ground into fine powder by a high speed disintegrator (YB-1000A, Yunbang Instrument Co., Ltd., Zhejiang, China). To remove lipids, pigments and other impurities, the powder was extracted three times with anhydrous ethanol at 60 °C, each for 5 h. Then, the purified sample powder was filtrated and air dried for further utilization.

#### 3.2.1. Single Factor Experiments

The polysaccharides of *R. minima* roots (PRM) was extracted using an ultrasound-assisted extraction method. Three major influencing factors (ultrasound exposure time, ratio of water to material, ultrasound extraction temperature) were selected for this experiment. The effects of these three factors on the yield of PRM were studied by a single factor design. Briefly, each single factor experiment was performed separately. The ultrasound exposure time range was from 5 to 50 min, while the ratio of water to material range was from 10 to 60 mL/g, and the ultrasound extraction temperature ranged from 40 to 80 °C. When one factor was changed, the other factors were left unchanged in each experiment. The effect of each factor was measured by determining the yield of PRM. The extraction yield (%) of PRM was determined by the phenol-sulfuric acid method [23] and calculated using the following equation:

(2) $$\text{PRM yield }(\text{Y},\%)=\frac{\text{C}\times \text{V}\times \text{N}}{\text{W}}\times 100$$

where C is the concentration of polysaccharide calculated from the calibrated regression equation (μg/mL); V is the total volume of extraction solution (mL); N is the dilution factor; and *W* is the weight of *R. minima* roots powder (μg).

#### 3.2.2. BBD and Statistical Analysis

In the present evaluation, the Design Expert Package (Version 8.0.5b, 2010, Stat-Ease Inc., Minneapolis, MN, USA) was employed for the experimental design, model building and regression analysis. According to the single factor experiment results, the best conditions of each variable, including ultrasound exposure time (A), the ratio of water to material (B) and extraction temperature (C), on the yield of PRM (Y) was confirmed, and then BBD was used to obtain the optimal extraction conditions for the extraction of PRM. These three factors were studied at three levels, coded −1, 0 and +1 for low, intermediate and high values, respectively. The levels of independent variables and the experimental runs for BBD are shown in Table 1. Seventeen runs based on BBD with three center points were carried out. To predict the optimized conditions, a second-order polynomial model was used to reflect the effect of the three independent variables on the yield of PRM [24]:

(3) $$\text{Y}=\beta_{0}+\sum_{\text{i}=1}^{3}\beta_{\text{i}}{\text{X}}_{\text{i}}+\sum_{\text{i}=1}^{3}\beta_{\text{ii}}{\text{X}}_{\text{i}}^{2}+\sum_{\text{i}=1}^{3}\sum_{\text{j}=\text{i}+1}^{3}\beta_{\text{ij}}{\text{X}}_{\text{i}}{\text{X}}_{\text{j}}$$

where Y is the response variable; β_0_, β_i_, β_ii_ and β_ij_ are the regression coefficients for intercept, linearity, square and interaction, respectively; X_i_ and X_j_ are the independent variables (i ≠ j), respectively.

The obtained data was analyzed by the statistical module of the Design Expert software 8.0.5. The equations were validated by analysis of variance (ANOVA) analysis. Three-dimensional plots and their respective contour plots were drawn to determine the individual and interaction effects of the tested variables on the response. Additionally, the hump of the three-dimensional plots could be used to identify their respective optimal parameters.

### 3.3. Preparation of Purified Fractions

The extracts of *R. minima* roots were precipitated with a five-fold volume of anhydrous ethanol, deproteinized with Sevag solution (chloroform-butyl alcohol, 4:1), and re-precipitated with anhydrous ethanol for 12 h at 4 °C. The precipitate was collected, freeze-dried, and named crude PRM. The crude PRM was further purified by DEAE-52 cellulose and Sephadex G-150 column chromatography. As a result, three fractions were obtained and coded as PRM1, PRM3 and PRM5. Each fraction was collected, concentrated, and dried for further use.

### 3.4. Monosaccharide Composition of Purified Fractions

The monosaccharide compositions of the purified fractions were analyzed by a HPLC method [25]. Briefly, 10 mg samples were hydrolyzed with 3 mL of 2 mol/L trifluoroacetic acid (TFA) at 100 °C for 8 h in a sealed tube. The hydrolysates were vacuum evaporated to remove the excess acid with methanol. Then, they were derivatized with 300 μL of 0.5 mol/L 1-phenyl-3-methyl-5-pyrazolone (PMP) under alkaline conditions. The monosaccharide derivatives were analyzed by a Waters e2695 HPLC system (Waters Co., Milford, MA, USA), equipped with a reverse phase C_18_ column (250 mm × 4.6 mm, 5 μm, Waters Co.), and detected by UV–Vis DAD detector. The PMP derivatives (10 μL) were injected and eluted with 83% phosphate buffer (0.1 mol/L, pH 6.7) and 17% acetonitrile (*v*/*v*) at a flow rate of 1.0 mL/min at room temperature. The UV detection wavelength was 245 nm.

### 3.5. In Vitro Antioxidant Activity of Purified Fractions

#### 3.5.1. Assay of DPPH Radical Scavenging Activity

DPPH radical scavenging activity was evaluated according to a previously described method [26]. Briefly, 50 μL of PRM1, PRM3 and PRM5 solutions (0.0625, 0.125, 0.25, 0.5, 1, 2, 4 and 8 mg/mL) were incubated with 150 μL of 0.3 mmol/L DPPH methanol solution for 20 min at room temperature. Then, the absorbance was read at 517 nm (SpectraMax M5, Molecular Devices, Sunnyvale, CA, USA). The DPPH radical scavenging activity was calculated by the following equation. The half maximal inhibitory concentration (IC_50_) value was determined from their dose-response curve:

(4) $$\text{DPPH radical scavenging activity }(\%)=\frac{{\text{A}}_{\mathrm{DPPH}}-{\text{A}}_{\mathrm{sample}}}{{\text{A}}_{\mathrm{DPPH}}}\times 100$$

where A_DPPH_ is the absorbance of the DPPH radical solution without sample and A_sample_ is the absorbance of the DPPH radical solution with the tested samples.

#### 3.5.2. Assay of Reducing Power

Reducing power was measured according to a previous method [27]. Briefly, 50 μL of PRM1, PRM3 and PRM5 solutions (0.0625, 0.125, 0.25, 0.5, 1, 2, 4 and 8 mg/mL) were incubated with 50 μL of 0.2 mol/L phosphate buffered saline (pH 6.6) and 50 μL of 1% potassium ferrocyanate (*w*/*v*) for 20 min at room temperature. Then, 50 μL of 10% trichloroacetic acid, 10 μL of 0.1% ferric chloride and 400 μL distilled water were added to the mixture. After 20 min, the absorbance was read at 700 nm (SpectraMax M5).

### 3.6. In Vitro Anticancer Activity of Purified Fractions

The human breast cancer MCF-7 cells were obtained from the American Type Culture Collection (ATCC, Manassas, VA, USA) and cultured in DMEM culture solution (10% fetal bovine serum, 100 U/mL penicillin, and 100 μg/mL streptomycin) with 5% CO_2_ at 37 °C. The *in vitro* anticancer activities of PRM1, PRM3 and PRM5 were measured by the MTT colorimetric method [28]. MCF-7 cells were suspended and seeded in 96-well plates and incubated for 24 h at a concentration of 5000 cells/well. PRM1, PRM3 and PRM5 solutions (0.0625, 0.125, 0.25, 0.5, 1, 2, 4 and 8 mg/mL) were added into the wells. After incubation for 24 h and 48 h, respectively, 100 μL MTT (5 mg/mL) was added and incubated for 4 h at 37 °C. Then, culture media were removed and 100 μL DMSO was added to each well. Absorbance was measured at 570 nm using a microplate reader (SpectraMax M5). The inhibitory rate of cell growth was calculated according to the formula:

(5) $$\text{Inhibitory rate }(\%)=\frac{{\text{A}}_{\mathrm{control}}-{\text{A}}_{\mathrm{sample}}}{{\text{A}}_{\mathrm{control}}}\times 100$$

where A_sample_ and A_control_ are the absorbance of groups with and without purified fractions treatment, respectively.

### 3.7. Statistical Analysis

Results were expressed as means ± standard deviation (SD) and each experiment was repeated three times. The difference was tested by one-way ANOVA, *p* < 0.05 was considered to be statistically significant.

## 4. Conclusions

In the present study, a rapid and sensitive ultrasound-assisted extraction technique was used to extract polysaccharides from *R. minima* roots. During this procedure, BBD was simultaneously applied to optimize the extraction parameters. The following optimum ultrasound-assisted extraction conditions were obtained: ultrasound exposure time, 21 min; ratio of water to material, 46 mL/g; ultrasound extraction temperature, 63 °C. Under these conditions, the experimental yield of PRM was 16.95% ± 0.07%. Moreover, all purified polysaccharide fractions had similar monosaccharide compositions. PRM3 and PRM5 exhibited potent antioxidant activities *in vitro* against DPPH radicals in a concentration-dependent manner. Purified fractions also showed reasonable anticancer activity *in vitro* against human breast cancer MCF-7 cells in a dose-dependent manner. This study provides a convenient and efficient extraction method for isolating polysaccharides from *R. minima* roots. In addition, it indicates that PRM3 can be developed as a potential antioxidant and active anticancer ingredient for the functional foods and pharmaceutical industries.
